# Drug interaction leading to prolonged sedation in a postoperative high risk coronary bypass surgery patient

**DOI:** 10.4103/0972-5229.78230

**Published:** 2011

**Authors:** Shrikant Bobade, Vinay Kulkarni, Sudhir Dhumne, Saurabh Barde, Jitesh Chauhan, Avinash Sharma

**Affiliations:** **From:** Department of Anaesthesiology and Critical Care, CARE Hospital, Nagpur, India

**Keywords:** Erythromycin, midazolam, prolonged sedation

## Abstract

Use of midazolam infusion in mechanically ventilated patient is an established practice in critical care. In our case, the use of erythromycin as a prokinetic agent for better tolerance of enteral feeding and paralytic ileus led to an interaction between midazolam and erythromycin, which resulted in prolonged and deeply sedated patient. In a critically ill patient, there is always a possibility of multiple drug interactions. It is important to understand them and they should be considered before starting new medication.

## Introduction

Drug interactions in critical care setting are of utmost importance. We report an interesting case of prolonged sedation due to erythromycin and midazolam interaction in a post cardiac surgery critically ill patient.

## Case Report

A 66-year-old male patient, who was a known case of hypertension, old cerebrovascular episode, post lumbar laminectomy with restricted mobility due to bilateral knee osteoarthritis, presented with history of unstable angina, 1 day prior to admission. His electrocardiogram confirmed anterolateral ischemia showing ST-T segment depression in anterolateral precordial leads V_2_–V_6._ His coronary angiogram revealed thrombus in left main coronary artery, left anterior descending artery (LAD) showing 99% occlusion, and right coronary artery showing 90% occlusion. A preoperative transthoracic echocardiography showed normal left ventricular systolic function (ejection fraction 60%). An urgent coronary bypass graft surgery was planned. Other comorbidities included benign prostatic hypertrophy and chronic constipation (able to pass motions only one to two times a week).

The patient underwent off-pump coronary artery bypass surgery with grafts to LAD, obtuse marginal branch of left circumflex coronary artery and posterior descending branch of right coronary artery. Intraoperative anesthesia management included midazolam 5 mg, fentanyl 500 μg, and vecuronium and sevoflurane 2–2.5%. Total urine output was 400 ml. The total duration in the operating theater was 5 hours. The patient was extubated at 7 hours after surgery in the recovery unit. The patient was stable on postoperative day 1, tolerating oral diet, and was ambulated out of bed.

On the 2^nd^ postoperative day, the patient was noted to have abdominal distension with weak peristaltic activity and tachycardia 120/minute. He was off inotropic support with mean arterial pressure of 70–80 mmHg and central venous pressure of 5–7 mmHg. Nasogastric tube aspiration drained 1.4 l/24 hour of bilious fluid, suggesting decreased gastrointestinal (GI) motility.

For improving the GI motility and tolerance of enteral feeding, serum potassium was optimized between 4.0 and 4.5 mEq/l; IV metoclopramide 10 mg 12 hourly, Tab. itopride 50 mg once daily and Tab. erythromycin 250 mg 6 hourly were started. Flatus tube was inserted for relieving abdominal distention.

Despite prokinetic therapy, ileus persisted and significant abdominal distention led to respiratory distress, tachycardia and appearance of hypoxemia. Serum creatinine increased to 2.01 mg/dl.

Patient had to be intubated on the 4th postoperative day and ventilated for worsening respiratory distress and hemodynamic instability. Circulatory support (noradrenaline infusion) was started and midazolam infusion commenced at 2–3 mg/hour for sedation. Midazolam infusion was stopped the next morning in view of weaning from ventilation. The patient received midazolam at a cumulative dose of 46 mg (15 hours). The patient remained sedated but arousable with no focal neurologic signs. Supportive therapy was continued (ceftriaxone-sulbactum, enoxaparin 40 mg once daily, aspirin–clopidogrel, erythromycin, itopride, metronidazole and pantoprazole).

GI ileus steadily improved by the 6th postoperative day in the form of decreased nasogastric aspirate and passage of feces. On postoperative day 6, he remained unresponsive with intact brainstem reflexes and normal electrolytes. Differential diagnosis included were metabolic encephalopathy, cerebrovascular event or prolonged midazolam effect. Intravenous flumazenil (antidote for midazolam) 200 mcg bolus was diagnostic of prolonged midazolam effect. Hence, computed tomography study of brain could be avoided. A possibility of prolonged midazolam effect due to erythromycin was entertained and Tab. erythromycin was stopped. Enteral feeding was established on 7^th^ day postoperatively.

Following satisfactory arousal from sedation, the patient was extubated on day 8 postoperatively. Further recovery remained uneventful and the patient was discharged home on day 15 postoperatively.

## Discussion

It is not very uncommon to notice paralytic ileus in an elderly patient, especially after cardiac surgery.[1] These patients with limited organ reserves pose challenges in the management of sedation for mechanical ventilation. Midazolam is an established sedative agent in intensive care due to its hemodynamic stability. When used as an infusion, it may have prolonged action due to cumulative effect.[2] We chose midazolam over propofol in this patient in view of his hemodynamic instability.

Unexpected delay in waking up after stopping midazolam infusion evokes suspicion of electrolyte imbalance, cerebrovascular episode or prolonged sedation due to midazolam. Probability of prolonged sedation due to midazolam was confirmed by awakening after flumazenil injection.

Erythromycin 250 mg 6–8 hourly has been shown to improve gastrointestinal motility and increase tolerance to enteral feeding in intensive care.[3]

Erythromycin and midazolam both are substrates for the same enzyme, cytochrome P450 IIIA4. So, there is competition for enzyme sites and metabolism of midazolam is inhibited leading to elevated levels and delayed elimination.[4]

This has been demonstrated clinically in cross-over studies, in which in patients receiving erythromycin, the effect of single dose midazolam was prolonged for 6 hours.[5,6]

Loss of consciousness following intravenous erythromycin administration was reported in an 8-year-child who underwent adenoidectomy due to interaction with oral midazolam administered as premedication.[7]

We believe that in our patient, the presence of erythromycin therapy was responsible for delayed arousal from midazolam and prolonged mechanical ventilation.

Drug interactions in critically ill patients, such as midazolam–erythromycin, can increase morbidity by prolonged sedation and delayed weaning from the ventilator. So, we suggest that concomitant use of macrolide antibiotics and midazolam should be avoided.
